# Clinical features and laboratory findings first case of B. 1.617.2 (delta) variant concern (VOC) in Iraq

**DOI:** 10.1016/j.amsu.2021.102814

**Published:** 2021-09-04

**Authors:** Rawand A. Essa, Sirwan K. Ahmed, Dunya H. Bapir, Shero A. Rasul, Awat A. Khdir, Chawan P. Abubakr

**Affiliations:** a Ph.D. in Cardiothoracic and Vascular Surgery, Lecturer in the University of Raparin, College of Nursing, Department of Adult Nursing, Rania, Sulaimani, Kurdistan-region, Iraq; b European Society for Thoracic Surgery (ESTS) Active Member, Iraq; c Department of Adult Nursing, College of Nursing, University of Raparin, Rania, Sulaimani, Kurdistan-region, Iraq; d Rania Teaching Hospital, Rania, Sulaimani, Kurdistan-region, Iraq; e Department of Medical Laboratory, College of Science, University of Raparin, Kurdistan-region, Iraq; f Rania Medical City Private Hospital, Rania, Sulaimani, kurdistan-region, Iraq; g Department of Critical Care Nursing, College of Nursing, Urmia University of Medical Science, Iran; h Rania Pediatric & Maternity Teaching Hospital, Rania, Sulaimani, Kurdistan-region, Iraq

**Keywords:** Variant concern (VOC), B.1.617.2, Delta variant, COVID-19, SARS-CoV-2, Effectiveness COVID-19 vaccines, Clinical features delta variant (VOC), Coronavirus

## Abstract

Since the initial report of the severe acute respiratory syndrome (SARS CoV-2) in Wuhan, China, in 2019, the virus has constantly mutated, resulting in the appearance of novel variants. In December 2020, the B.1.617.2 (delta) variant concern (VOC) was first reported in India, and rapidly spread around the globe, is now the main brand in the United Kingdom, and it has grown dramatically. Here we present the clinical features and laboratory findings of the first case of B. 1.617.2 (delta) variant concern (VOC) in Iraq. A 6-year-old female child presented with severe abdominal pain, headache, severe vomiting, and diarrhea, runny nose, alerted mental status, loss of appetite, and fever. The patient was diagnosed with COVID-19 delta variant B.1.617.2 by RT-PCR. The patient was treated by administration of glucose saline 4% for 3 days, ceftriaxone vial 1 mg every 12 h for 6 days, and an acetaminophen bottle on a need to prevent fever followed by a Flagyl bottle every 24 h for 3 days. Vaccination and prevention the spread of the virus and against it are important preventive approaches for delta variant. Sore throat, runny nose, headache, and vomiting, diarrhea are the major clinical features of the delta variant. This was followed by an elevation of the leukocyte WBC, and blood platelets. To reduce the impact of new delta variant B.1.617.2 infection; handwashing, wearing a double mask, avoiding crowded and closed settings, social distancing, lockdown, and ensuring good ventilation are major significant options against this variant.

## Highlights

• Sore throat, runny nose, headache, vomiting, diarrhea are major clinical findings of delta variant concern (VOC) B.1.617.2.
• To reduce the impact of new delta variant B.1.617.2 infection; hand washing, wearing double mask, avoiding crowded and closed setting, social distancing, lockdown, and ensure good ventilation are major significant options against this variant.

## 1 Introduction

Since the initial report of the severe acute respiratory syndrome (SARS CoV-2) in Wuhan, China in 2019, the virus has constantly mutated, resulting in the appearance of novel variants [1,2]. SARS-CoV-2 has caused millions of deaths globally, producing thousands of variants spreading around the world [3]. COVID-19 was formerly thought to just cause respiratory dysfunction; however numerous clinical presentations have revealed that COVID-19 is a systemic disease not limited to the lungs [4,5]. The B.1.617.2 (delta) variant concern (VOC) was first reported in India in December 2020 and rapidly spread around the globe, and is now the main brand in the United Kingdom, France, Japan, and increasing dramatically [[6], [7], [8]]. The main symptoms of infection with the delta variant are sore throat, headaches, and runny nose. The WHO warns that the Delta variant is quickly becoming the main strain of SARS-CoV-2 worldwide, due to its higher transmissibility and mortality rate [[9], [10], [11]]. Globally, numerous treatment options have been examined for hospitalized patients with COVID-19 (e.g., antiviral agents [[12], [13], [14]], antimalarial drugs [15], glucocorticoids [12,16], convalescent plasma [17,18], and immunomodulators [12,[19], [20], [21], [22]]. Additionally, herbal treatment medicine was used against delta variant B.1.617.2 [23]. The world's largest purchase of the AstraZeneca vaccine, the efficacy of this vaccine against the delta variant is only %10.4 [24]. For this reason, the transmissibility of this variant was higher than of previous variants and, distressing. In Iraq, particularly in Kurdistan-region populations have a lack of information about this variant. Additionally, vaccine hesitancy, political, financial, and economic issues are major reasons to get vaccines. To date, numerous variants have been reported in different countries.

To the best of our knowledge, this is the first case of delta variant coronavirus in Iraq. The current study aims to present clinical features and laboratory findings of the first case delta variant B.1.617.2 in Iraq. The current paper has been written in the line with SCARE guidelines [25].

## 2 Case presentation

### 2.1 Patient information

On July 20, 2021, a 6-year-old female, was admitted to the Hajiawa General Hospital (HGH) government hospital in the Kurdistan region, Iraq, she presented with severe abdominal pain, headache, severe vomiting, and diarrhea, runny nose, alerted mental status, loss of appetite and fever with recurrent urinary tract infection (UTI). But with no past surgical history.

### 2.2 Clinical findings and diagnostic assessment

Subsequently, the patient^'^s temperature was 39,1 °C normal range (35.5 °C–37.5 °C), Blood pressure (BP) was 90/58 mmHg (hypotension), normal range (120/80 mmHg), respiratory rate was 13/min, pulse rate 115 bpm, and patient oxygen saturation (SPO2) was % 95 at rest without oxygen. The murphy sign was negative.

Furthermore, laboratory test results showed elevated leucocytes 23.7 × 10^9^/l (normal range 3.5–9.5 × 10^9^/l), lymphocyte 12.8 × 10^9^/l (normal range 1.1–3.2 × 10^9^/l), granulocyte 20.2 × 10^9^/l (normal range 1.1–8.1 × 10^9^/l), blood platelet 398 × 10^9^/l (normal range 125–350 × 10^9^/l), neutrophils 3.58 × 10^9^/l (normal range 1.8–6.3 × 10^9^/l, red blood cells (RBC) 4.54 × 10^12^/l) (normal range 125–350 × 10^12^/l) and C-reactive protein 101 mg/dl (normal range 0.00–0.60 mg/dl). In addition, the liver function test revealed that alkaline phosphate (ALP) 170 Lu/L normal range for male (40–130 Lu/L) and for female is (32–92 Lu/L), S.G.O.T(AST) 23Lu/L (normal range 7–46 Lu/L), S.G.P.T (ALT) 11 Lu/L (normal range7–49 Lu/L), serum amylase was 45 U/dl (normal range 25–125 U/dl), serum lipase was 25 U/dl (normal range 13–42 U/dl), serum total bilirubin was 0.2 mg/dl (normal range 0.3–1.2 mg/dl). The patient was negative para influenza viruses, respiratory adenovirus, and syncytial virus. In addition, on July 21, 2021 the patient was diagnosed with COVID-19 delta variant B.1.617.2 by RT-PCR, and genomic sequencing.

### 2.3 Therapeutic intervention

**Fig. 1:**
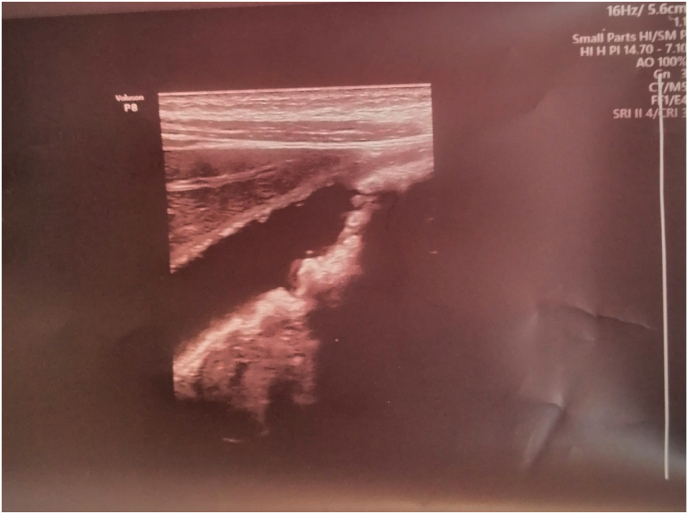
Multiple stones each around 2–4 mm gall bladder relates intravenous injection, such as ceftriaxone.

On July 17, 2021, a 6-year-old female immediately administer Glucose saline 4% for 3 days, ceftriaxone vial 1 mg every 12 hours for 6 days duration, acetaminophen bottle on the need to prevent fever followed by Flagyl bottle for every 24 hours for 3 days duration. On July 18, 20,201, the patient had severe diarrhea 30 times per day, with a fully runny nose. On July 20, 2021, the patient was referred to the radiologist for further examination. The ultrasound of abdominal and pelvic was performed revealed normal liver in size, shape, and echogenicity, normal spleen and pancreas, normal both kidneys in shape, size, and echogenicity with acceptable cortical thickness, no stone, hydronephrosis, hydroureter and normal urinary bladder.no fluid-filled in bowel loops and no features of appendicitis apart from multiple stones each around 2–4 mm gall bladder related intravenous injection, such as ceftriaxone (Fig. 1). However, the general urine examination was within normal limits. On July 23, 2021, the first author stopped the ceftriaxone vial due to side effects for children, such as cholelithiasis. On July 24, 2021, the patient feels better and removed the cannula. Three days later, the patient's RT-PCR was negative, and was discharged from the hospital.

### 2.4 Follow up

The patient was discharged for outpatient monitoring.

## 3 Discussion

The literature confirmed that COVID-19 has numerous variants, and complications globally [26]. The new delta variant B.1.617.2 rapidly spearing worldwide. Previously, the delta variant was detected in India, followed by the United Kingdom and the United States. B.1.617.2 bears spike mutations G142D, T19R, F157del, E156G, R158del, L452R, D614G, P681R, T478K, and D950 N relative to Wuhan-1 D614G 118 [27]. The main strength of our case did not travel history to countries with a high prevalence of delta variant B.1.617.2. Clinical features of our case are headache, sore throat, fever, runny nose, vomiting, diarrhea, loss of appetite, altered mental status. The laboratory findings of our case revealed that (elevated leucocytes WBC, granulocytes, lymphocytes, and blood platelets). Regarding the liver function test only elevated alkaline phosphate may be related to intravenous injection, such as ceftriaxone. However, significantly, only 6.5% of all COVID-19 cases occurred in fully immunized people, relatively few of these cases required admission to hospital [28]. In contrast, our case did not get the COVID-19 vaccine. In Iraq, there is a considerable lack of information about patients with COVID-19 caused by the delta variant compared to previous variants. However, the delta variant B.1.617.2 became globally [29]. Currently, there is no published research in Iraq about this variant. A recent study revealed that the estimated vaccine effectiveness against delta variant was approximately 88% with two doses of the BNT162b2 vaccine and approximately 67% with two doses of the ChAdOx1 nCoV-19 vaccine [30]. In another study found the effectiveness of the Pfizer vaccine was 79% (75–82) infected cases with delta variant concern (VOC), while the AstraZeneca vaccine, its effectiveness, approximately 60% (53–66) [31]. Moreover, Iraq has limited devices and preventive measures against this variant; for this reason, we suggest vaccination, wearing masks, social distancing, and lockdown [32,33] are the great challenges to reduce the impact of the delta variant B.1.617.2. the limitation of our study is only one case; therefore, a large sample size is required to established clinical features and laboratory findings of the B.1.617.2 delta variant concern (VOC) in Iraq. our case may be an early signal for delta variant expansion. Further study is required to know the variants of this virus in Iraq.

## 4 Conclusion

Vaccination and preventing the spread of the virus and against it are important preventive approaches of delta variant. Sore throat, runny nose, headache, vomiting, diarrhea are major clinical features of the delta variant. Followed by elevation of leucocytes WBC, and blood platelets. To reduce the impact of new delta variant B.1.617.2 infection; hand washing, wearing the double mask, avoiding crowded and closed settings, social distancing, lockdown, and ensure good ventilation are major significant options against this variant. Therefore, fully vaccinated people should wear the mask and practice COVID-19 preventive strategies.

## Author contribution

**Rawand A. Essa, Sirwan K. Ahmed:** Conception and design, execution, analysis and interpretation of data, involved in drafting the article, revised it critically for important intellectual content, read and approved the final version of the manuscript.

**Dunya H. Bapir**, **Shero A. Rasul, Awat A. Khdir, Chawan P. Abuabkr**: involved in drafting the article, revised it critically for important intellectual content, read and approved the final version of the manuscript.

## Funding

This research did not receive any specific grant from funding agencies in the public, commercial, or not-for-profit sectors.

## Ethical approval

Ethical approval has been given by the ethics committee of Rania Medical City (RMC).

## Consent

Written informed consent was obtained from the parents of this patient for publication of this case report and accompanying images. A copy of the written consent is available for review by the Editor-in-Chief of this journal on request.

## Guarantors

Dr. **Rawand A. Essa**, and Registered Nurse **Sirwan K. Ahmed**: Accept full responsibility for the work and conduct of the study, had access to the data, and controlled the decision to publish.

## Provenance and peer review

Not commissioned, externally peer-reviewed.

## Registration of research studies

Not applicable.

## Declaration of competing interest

The authors declare that they have no known competing financial interests or personal relationships that could have appeared to influence the work reported in this paper.
